# Technology-based interventions for tobacco smoking prevention and treatment: a 20-year bibliometric analysis (2003–2022)

**DOI:** 10.1186/s13011-024-00595-w

**Published:** 2024-02-06

**Authors:** Waleed M. Sweileh

**Affiliations:** Department of Biomedical Sciences, Faculty of Medicine and Health Sciences, An-Najah National University, Nablus, Palestine

**Keywords:** Substance abuse, Tobacco smoking, Technology-based, Text-messages, Smartphone apps, Research analysis

## Abstract

**Background:**

Substance abuse, particularly tobacco smoking, is a significant global public health concern. Efforts have been made to reduce smoking prevalence and promote cessation, but challenges, such as nicotine addiction, marketing tactics by tobacco industry, and cultural acceptability hinder progress. Technology has emerged as a potential tool to address these challenges by providing innovative scalable interventions. The objective of the study was to analyze and map scientific literature on technology-based intervention for tobacco prevention and treatment.

**Methods:**

A bibliometric methodology was conducted. Scopus database was used to retrieve relevant research articles published between 2003 and 2022. The analysis included publication trends, key contributors, research hotspots, research themes, the most impactful articles, and emerging research topics.

**Results:**

A total of 639 articles were found, with a slow and fluctuating growth pattern observed after 2011. The *Journal of Medical Internet Research* was the most prominent journal in the field. The United States was the leading country in the field, followed up by the United Kingdom, and the Netherlands. Research hotspots included smoking cessation, randomized controlled trials, and technology-based methods such as internet, mHealth, smartphone apps, text messages, and social media. Four primary research themes were identified: development of smartphone applications, efficacy of text messaging interventions, acceptance and effectiveness of smartphone applications, and interventions targeting young adults and students using mobile phone and social media platforms. The top 10 cited articles demonstrated effectiveness of digital interventions in promoting smoking cessation rates and reducing relapse rates. Emerging research topics included the use of virtual reality interventions, interventions for specific populations through personalized tools, and technology-based interventions in non-Western countries.

**Conclusions:**

The findings of the current study highlight the potential of technology to address the challenges associated with tobacco smoking. Further future research in this area is warranted to continue advancing the field and developing effective and evidence-based interventions to combat tobacco smoking.

**Supplementary Information:**

The online version contains supplementary material available at 10.1186/s13011-024-00595-w.

## Background

Substance abuse, particularly tobacco smoking, continues to be a significant public health concern worldwide [1]. Over the past three decades, tremendous governmental and non-governmental efforts have been implemented to curb smoking prevalence, aiming to decrease tobacco smoking, prevent initiation among youth, and promote smoking cessation among current smokers [1]. However, smoking is a highly addictive behavior due to the presence of nicotine, a potent stimulant and psychoactive substance [2, 3]. Furthermore, the tobacco industry has employed various marketing tactics to promote smoking. Such tactics include extensive advertising, attractive packaging, and innovative products with different flavors have attracted different target populations [4, 5]. The wide spread use of tobacco has also been attributed to the cultural and societal acceptability of smoking [6, 7]. The addictive properties of nicotine, the marketing tactics by tobacco industry, and cultural acceptability of smoking are the main challenges facing individuals who want to quit smoking. In addition, the addictive properties of nicotine is the main cause of the persistently higher prevalence rates of tobacco use [8, 9]. Nicotine stimulates the release of dopamine in the brain, creating a rewarding sensation and reinforcing the habit-forming cycle. Over time, regular smoking leads to nicotine dependence, making it difficult for individuals to quit without nicotine compensation [2, 10]. Despite all these difficulties, a wide range of important initiatives and preventive policies have been implemented nationally or internationally to tackle the challenges of smoking [11]. One important example of an international initiative to curb smoking is the World Health Organization (WHO) Framework Convention on Tobacco Control (FCTC), which stands as the first global treaty negotiated under the auspices of the WHO [1].

As societal behaviors and communication patterns have evolved, technology has emerged as a powerful tool with the potential to revolutionize the substance abuse prevention and treatment [12, 13]. Traditionally, substance abuse prevention and treatment methods have relied on conventional methods such as face-to-face counseling, group therapy, and printed educational materials [14, 15]. While these conventional methods have demonstrated some efficacy, they often face limitations in reach, accessibility, and engagement. However, with the rapid advancement of technology, new avenues have emerged, providing innovative ways to address substance abuse [16, 17]. For example, technology-based interventions can reach a wider audience by leveraging the pervasive nature of digital technologies. Technology can overcome geographical barriers and reach individuals living in remote and underserved areas provided that they have adequate digital literacy. Technology provides real-time monitoring and assessment, enabling personalized interventions tailored to an individual’s specific needs, with high degree of privacy reducing stigma and fear of judgment [18–20]. Furthermore, the interactive and immersive nature of technology allows for engaging and interactive interventions that can increase motivation and promote behavioral change. Therefore, technology can be more cost-effective and scalable compared to conventional methods [21–23]. Social media platforms are another important aspect of digital technology that offer a unique opportunity to reach and engage with large number of populations. Social media enables for the formation of online communities and support networks, allowing for peer and social support. It could be argued that social media could be used to encourage and promote smoking, especially among young generations through the portrayal of smoking in movies and tobacco industry advertisements. However, the platform used for the promotion of tobacco smoking are usually not the same platforms used to help smokers quit smoking and usually don’t use the same networks.

Research is considered a key element in building evidence-based preventive policies and strategies to curb persistent smoking rates. The WHO emphasizes the importance of research to address global health challenges [24]. Similarly, the US CDC supports research on health promotion and preventive strategies [25]. The National Institutes of Health (NIH) provide funding and resources for research on combating health challenges. Recognizing the addictive nature of tobacco smoking, advantages of digital technology, and the recommendations by international health organizations to research major global health challenges, research efforts have focused on implementing technology to reduce tobacco smoking rates through preventive and treatment interventions. Based on all the above-mentioned information, it is imperative to do a comprehensive analysis of research on prevention and treatment of tobacco smoking using digital technologies. Therefore, the current study aims to analyze and map scientific literature on technology-based interventions for the prevention and treatment of tobacco smoking. The study aims to achieve the following objectives: (1) identify research volume, growth pattern, and key contributors to the field; (2) identify the main research hotspots and research themes in the field; (3) analyze the content of the top 10 cited articles in the field; and (4) identify the emerging research topics in the field.

## Method

### Database

To analyze and map the research landscape, a comprehensive methodology was developed and implemented in Scopus database. The Scopus (www.scopus.com) has several advantages regarding the number and type of indexed journals as well as features related to handling and export of data for analysis and mapping.

### Keywords

The study focused on retrieving relevant research articles from the Scopus database using a set of specific keywords related to digital technologies including social media platforms in addition to keywords related to tobacco smoking. All keywords were used in the title search to minimize false-positive results. Supplement 1 shows the keywords used and the inclusion and exclusion criteria.

### Inclusion and exclusion criteria

The followings were used as inclusion criteria: (1) research articles published between 2003 and 2022; (2) English language articles; and (3) Quantitative, qualitative, and mixed-methods studies. The followings were used as the exclusion criteria: (1) review articles, editorials, commentaries, and letters to the editor; (2) studies that do not specifically focus on traditional tobacco smoking such as e-cigarettes, electronic cigarettes, and e-cig.

### Validation

**Fig. 1:**
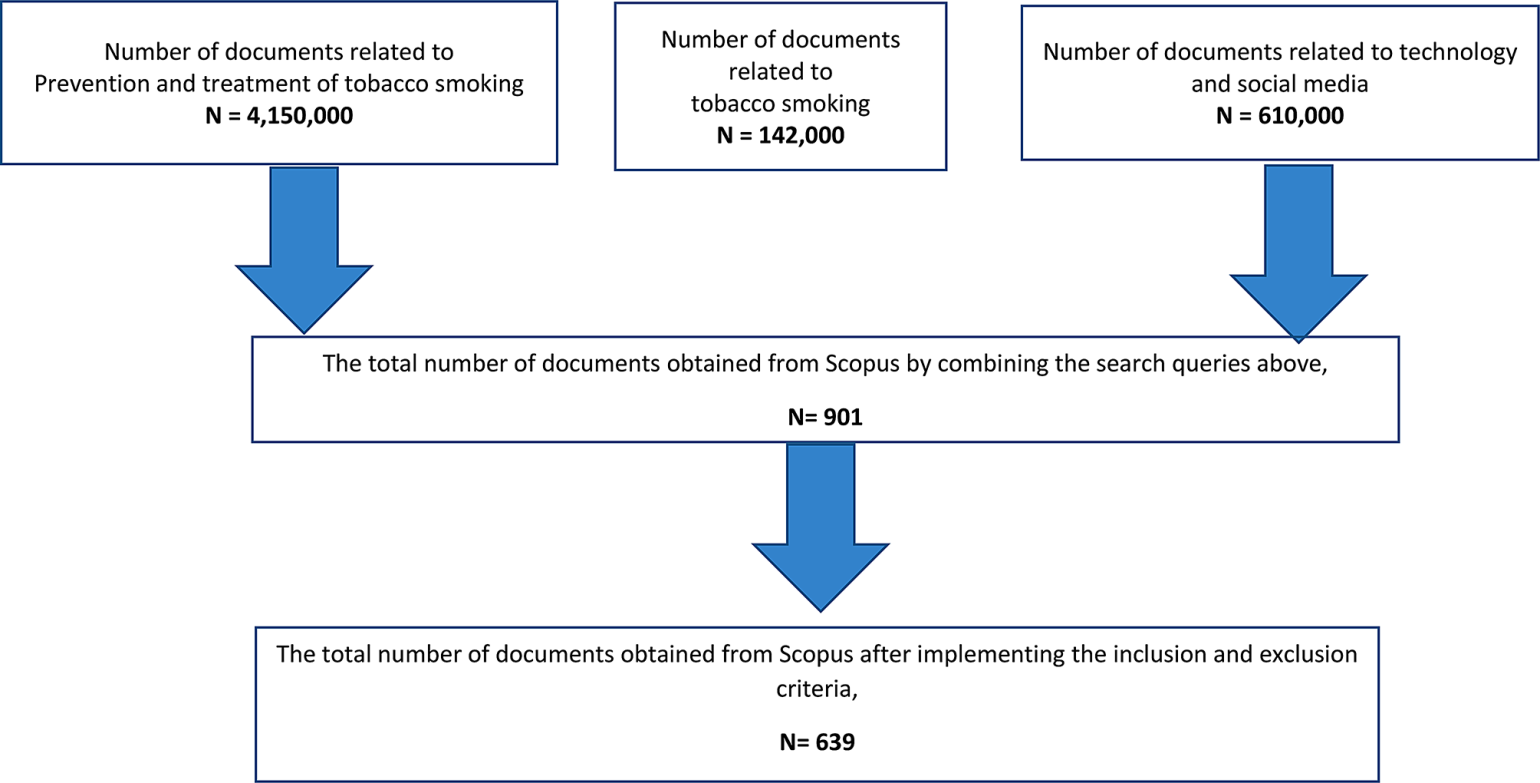
Flow chart for the search strategy on technology-based interventions for tobacco smoking prevention and treatment

The retrieved articles underwent a rigorous validation process to ensure their relevance and accuracy. A validation sample comprising 100 retrieved articles was carefully examined by two knowledgeable colleagues in the field of medicine. Based on their expertise, it was strongly recommended to include two additional specific terms, namely opioids and e-cig in the exclusion step. This crucial addition was deemed necessary to mitigate the risk of false-negative results and enhance the overall validity of the study. The number of documents retrieved in each step in the search strategy is shown in Fig. 1.

### Data analysis and visualization

The retrieved articles were analyzed quantitatively to gain insights into the research landscape. Key metrics such as publication trends, journal, country, and author distributions were examined to understand the research output. VOSviewer [26, 27], a widely used free online program, was used to create maps for the identification of most frequent author keywords (research hotspots) and research clusters (research themes).

### Interpretation of VOSviewer maps

We undertook a rigorous mapping analysis of the retrieved dataset using VOSviewer. In VOSviewer maps, each term or keyword in the dataset is represented as a point on the map, denoted by a circle or node. These nodes vary in size and color, and they are interconnected by lines of differing thickness. The size of a node serves as an indicator of the term’s significance or prevalence within the dataset. Larger nodes signify that a specific term is frequently discussed or holds a central role in the body of research, while smaller nodes denote less commonly mentioned concepts. The colors assigned to these nodes facilitate the grouping of terms into thematic clusters, with terms of the same color typically belonging to the same cluster or sharing a common thematic thread. The spatial proximity of nodes on the map reflects their similarity in meaning or concept. Nodes positioned closely together share a robust semantic or contextual connection and are likely to be co-mentioned in research articles or share a similar thematic focus. Conversely, nodes placed farther apart suggest a lower degree of commonality in terms of their usage in the literature. The lines that link these nodes represent the relationships between terms. The thickness of these lines provides insights into the strength and frequency of these connections. Thick lines indicate that the linked terms are frequently discussed together or exhibit a robust thematic association, while thinner lines imply weaker or less frequent connections. In essence, VOSviewer maps offer a visual narrative of the underlying structure and relationships within your dataset. By examining node size and color, we can identify pivotal terms and thematic clusters. Simultaneously, analyzing the distance between nodes and line thickness unveils the semantic closeness and strength of associations between terms. These visual insights are invaluable for researchers seeking to unearth key concepts, identify research clusters, and track emerging trends within their field of study.

### Emerging research topics

To uncover the current trends in research within the field, we conducted an analysis of 65 articles published in 2022. Through the use of VOSviewer, we created a visualization and interpretation of the articles, allowing us to deduce the prominent research topics and emerging areas of interest.

## Results

### Volume, growth pattern, and key contributors

**Fig. 2:**
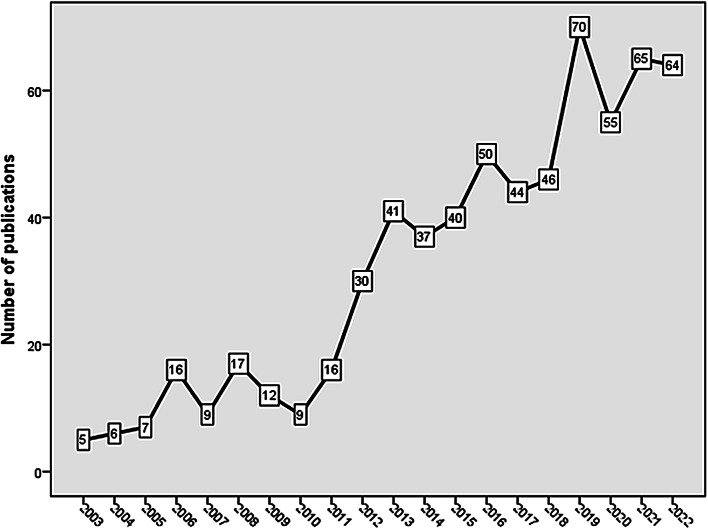
Annual growth of publications on technology-based interventions for tobacco smoking prevention and treatment

The search strategy yielded 639 pertinent articles spanning from 2003 to 2022. The annual growth of publications revealed a slow and upward fluctuating growth after 2011 (Fig. 2). The *Journal Of Medical Internet Research* ranked first (*n* = 73; 11.4%), followed by the *Nicotine and Tobacco Research* journal (*n* = 53; 8.3%), and *JMIR Mhealth and Uhealth* (*n* = 37; 5.8%), and *BMC Public Health* journal (*n* = 23; 3.6%). The results indicated a significant contribution from the United States (US) (*n* = 359; 56.2%), followed by the United Kingdom, the Netherlands, Australia, Canada, and Germany. It is noteworthy that China ranked seventh and India ranked eleventh. Scholars from 51 different countries have made contributions to the field of technology-based smoking prevention and treatment. However, of these 51 countries, only 13 made a significant contribution of 10 articles or more each. Contribution of countries in the Middle East, Africa, Latin America, East Europe, and certain areas in Asia was relatively low. Upon analyzing the research activity, it was found that certain institutions have made notable contributions to the topic. Leading the pack is the Universiteit Maastricht (the Netherlands) (*n* = 36; 5.6%), followed by the University of California, San Francisco (United States), Brown University (United States), and Fred Hutchinson Cancer Research Center (United States). The National Cancer Institute (*n* = 141; 21.7%) of the US NIH ranked first as a funding sponsor for the retrieved articles. A total of 3709 authors collaborated in publishing the 639 research articles that were found. On average, each article had 5.8 authors. There were 15 (2.3%) articles with a single author, 41 (6.4%) with two authors, 62 (9.7%) with three authors, and 102 (16.0%) with four authors each. The remaining 419 (65.6%) articles were multi-authored (≥ 5). The most prolific author was Graham, (A) L. (*n* = 30; 4.7%), followed by De Vries, H (*n* = 28; 4.4%), and Bricker, J. (B) (*n* = 20; 3.1%).

### Research hotspots and research themes

**Fig. 3:**
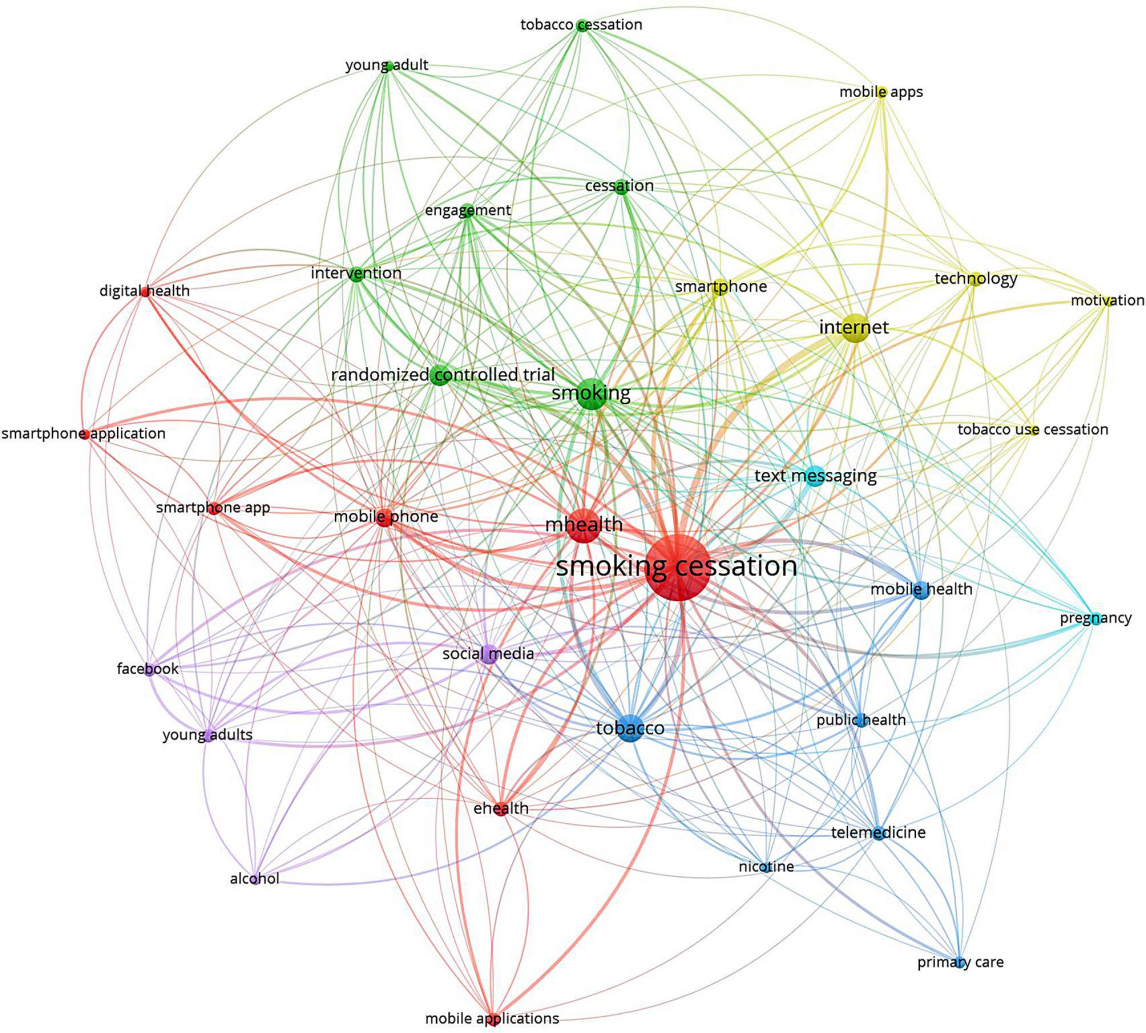
Mapping frequent author keywords to identify research hotspots on technology-based interventions for tobacco smoking prevention and treatment

When visualizing author keywords that appeared at least 10 times, a map consisting of 33 keywords were generated (Fig. 3). The “smoking cessation” keyword had the largest node size suggesting that the majority of the retrieved literature focused on smoking cessation research efforts [26, 27]. The map included smaller nodes for the keywords “randomized controlled trial”, adolescence, young adults, and pregnancy. The map included different technology-based methods used in the context of prevention and treatment of smoking, with “internet”, “mHealth”, “smartphone app.”, “text message”, and ‘social media” being the most frequently mentioned.

**Fig. 4:**
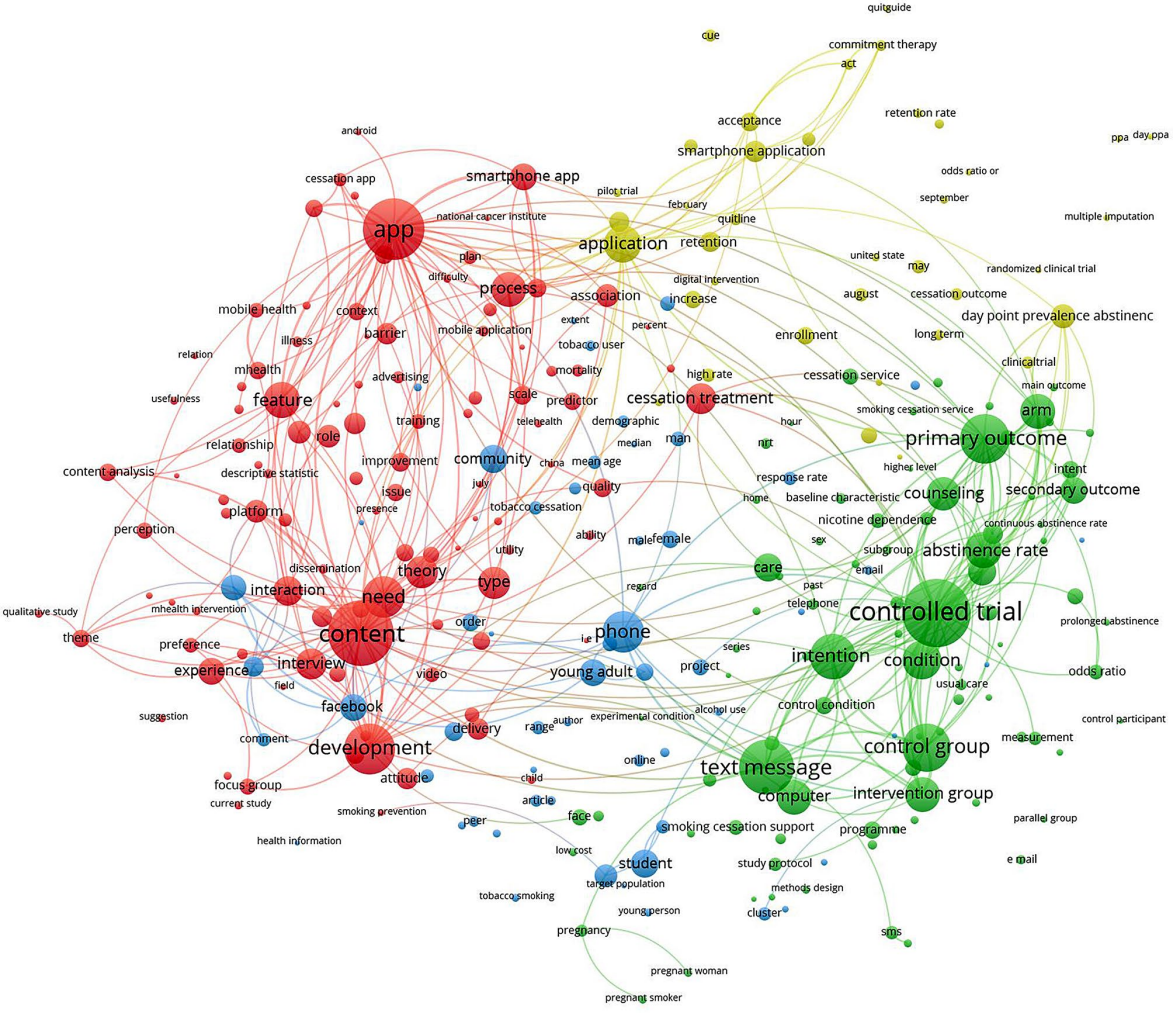
Mapping frequent terms in titles and abstracts to identify research themes on technology-based interventions for tobacco smoking prevention and treatment

The visualization map derived from the frequent terms in titles and abstracts of the retrieved articles revealed four primary research themes represented as four clusters with different distinct colors (Fig. 4). The red cluster signifies a prominent research focus on the development and content of smartphone applications designed for smoking cessation purposes [28, 29]. The green cluster represents research predominantly centered around Randomized Clinical Trials (RCTs) aimed at assessing the efficacy of text messaging interventions for smoking cessation therapy [30, 31]. The yellow cluster denotes research investigations pertaining to the acceptance and the effectiveness of smartphone applications in supporting smoking cessation efforts [32–35]. Lastly, the fourth cluster encompasses research-related to technology-based interventions utilizing mobile phones and social media platforms targeting smoking cessation among young adults and students [36–38].

### The most impactful articles

In the present study the top ten cited articles in the field were analyzed. These highly influential studies have played a pivotal role in substantiating the efficacy of digital interventions in augmenting smoking cessation rates and ameliorating relapse rates. Each of these articles has contributed valuable insights into the field, and their findings collectively provide a strong foundation for the effectiveness of digital approaches in addressing smoking cessation and relapse prevention. Their findings offer invaluable insights for healthcare professionals, policymakers, and researchers, and their consistent trends affirm the efficacy and potential of digital approaches in smoking cessation and relapse prevention.

The criteria employed to evaluate the efficacy of these digital interventions have been diverse, reflecting a comprehensive assessment of their impact. Some common criteria include the measurement of smoking cessation rates, often assessed through 7-day point prevalence abstinence or continuous abstinence, and, in some cases, employing biochemical verification to confirm participants’ smoking status [39, 40]. Long-term abstinence, often measured at 6 months or 12 months post-cessation, has been a key indicator of sustained efficacy [40–42]. User engagement with digital interventions has also been evaluated, with studies showing that higher engagement often correlates with better outcomes [43–45]. Comparative studies have examined the advantages of digital approaches in contrast to traditional methods such as counseling or pharmacotherapy [42, 46, 47]. Cost-effectiveness has been assessed, shedding light on the economic benefits of these interventions [48–50].

Table 1 shows the top 10 articles that received the highest number of citations [44, 51–59]. In summary, the articles demonstrated the effectiveness of digital interventions such as mobile phone text messaging, smartphone applications, web-based programs, and social media-based interventions in promoting smoking cessation rates and reducing relapse rates. The interventions have shown higher quit rates and increased continuous abstinence rates compared to control groups. Participants reported high satisfaction and perceived the interventions helpful in their quit attempts. The article that received the highest number of citations (*n* = 576) was a RCTs called “txt2stop” and used mobile phone text messaging [57]. The trial showed significant successful smoking cessation rates and the author suggested inclusion of the method in smoking cessation services. The second highly cited article “Do u smoke after txt” showed similar effectiveness in smoking quit rates [59]. The third highly cited articles was a web-based computer tailored smoking cessation program as a supplement to nicotine patch therapy [52]. The trial showed a significantly higher abstinence rates in the interventional group compared to control group. The fourth cited article was about the development of a program designed to deliver text messages to help college students quit smoking [56]. The program was tested in a small number of college students and showed promising results that support the use of wireless messages in smoking cessation services. In a study that aimed to evaluate popular smartphone apps designed to help people quit smoking, authors revealed that these apps had limited adherence to clinical guidelines. This underscores the need for improving app content and design to better align with evidence-based practices [29]. Another study explored how user engagement affected the effectiveness of a web-based smoking cessation program [60]. The results indicated that greater user engagement, as measured by the number of web sections opened, was associated with a higher likelihood of quitting smoking. However, certain demographic factors, like age, gender, and education level, were linked to lower engagement, especially when program sections were presented sequentially. Another study provided an early assessment of QuitNet, a widely accessible online smoking cessation website [61]. Researchers revealed a 7% point prevalence abstinence rate among QuitNet users, which increased to 30% when considering only those who responded to the survey. The study shed light on the potential impact of web-based behavior change programs for smoking cessation. It underscored the critical role of user engagement and support in achieving positive outcomes.

**Table 1:** The top 10 cited articles on technology and social media-based tobacco smoking prevention and treatment

Title	Objective	Digital Tool Investigated (intervention)	Finding and Conclusion	Number of Citations
Smoking cessation support delivered via mobile phone text messaging (txt2stop): A single-blind, randomised trial	Assess the effectiveness of a mobile phone text messaging smoking cessation program	Mobile phone text messaging (txt2stop)	The txt2stop smoking cessation program significantly improved smoking cessation rates at 6 months and should be considered for inclusion in smoking cessation services	576
Do u smoke after txt? Results of a randomised trial of smoking cessation using mobile phone text messaging	Determine the effectiveness of a mobile phone text messaging smoking cessation program	Mobile phone text messaging	The program offers potential for helping young smokers quit, being affordable, personalized, and age-appropriate	572
Randomized controlled trial of a web-based computer-tailored smoking cessation program as a supplement to nicotine patch therapy	Assess the efficacy of web-based tailored behavioral smoking cessation materials among nicotine patch users	Web-based tailored materials	Web-based tailored materials were more effective in achieving continuous abstinence compared to non-tailored materials	254
Randomized, controlled pilot trial of a smartphone app for smoking cessation using acceptance and commitment therapy	Test the feasibility, acceptability, preliminary efficacy, and mechanism of change of a smartphone-delivered acceptance and commitment therapy (ACT) application for smoking cessation	Smartphone-delivered ACT application	SmartQuit showed higher engagement and promising quit rates compared to an application following US Clinical Practice Guidelines	249
A content analysis of popular smartphone apps for smoking cessation	Examine the content of popular smartphone apps for smoking cessation	Smartphone applications (apps)	Popular apps have low levels of adherence to clinical guidelines for treating tobacco use and dependence	247
College smoking-cessation using cell phone text messaging	Evaluate a prototype program targeting college students that integrates Web and cell phone technologies for smoking cessation	Cell phone text messaging	Support for using wireless text messages to deliver potentially effective smoking-cessation behavioral interventions to college students	243
Happy Ending: A randomized controlled trial of a digital multi-media smoking cessation intervention	Assess the long-term efficacy of a fully automated digital multi-media smoking cessation intervention	Digital multi-media smoking cessation intervention	The digital multi-media intervention showed higher repeated point abstinence rates and improved adherence to NRT	226
Initial evaluation of a real-world internet smoking cessation system	Evaluate the results of a broadly disseminated smoking cessation website (QuitNet)	Web-based smoking cessation website	QuitNet demonstrated benefits for smoking cessation, with sustained use and social support enhancing the outcomes	205
The role of engagement in a tailored web-based smoking cessation program: Randomized controlled trial	Investigate whether engagement in a web-based smoking cessation program predicts 6-month abstinence	Web-based smoking cessation program	Engagement with the program is a key mechanism underlying the impact of web-based smoking cessation interventions	202
Web-Based Smoking-Cessation Programs. Results of a Randomized Trial	Identify active psychosocial and communication components of a web-based smoking-cessation intervention and examine the impact of increasing the tailoring depth	Web-based smoking-cessation program	High-depth tailored success stories and high-personalized message source were most influential in achieving cessation, and tailoring depth had a positive effect on quit rates	201

### Emerging research topics

The screening process involved analyzing articles published in 2022 to uncover emerging research topics concerning the technology-based prevention and treatment of tobacco smoking. While some of the identified research topics were extensions of ongoing investigations that began a decade ago, there were also a few novel areas of exploration. To identify the emerging research topics on technology-based interventions for prevention and treatment of tobacco smoking, articles published in 2022 were screened. Research topics identified in the articles published in 2022 (*n* = 65) were presented in Table 2. The following research topics are considered emerging research topics:

**Table 2:** Research topics present in articles on technology-based tobacco smoking prevention and treatment; published in 2022

Research topic	Number of articles	(%) *N* = 65
Mobile phone/text message interventions for smoking cessation	15	23.1
Evaluation of the effectiveness and outcomes of technology-based smoking cessation programs	12	18.5
Use of technology-based interventions for smoking cessation in different countries or regions (e.g., Japan, Brazil, Thailand, South Africa)	10	15.4
Smoking cessation interventions for specific populations (e.g., socioeconomically-disadvantaged young adults, HIV patients, cancer survivors, veterans)	8	12.3
Smartphone applications for smoking cessation	7	10.8
Telemedicine/telehealth interventions for smoking cessation	5	7.7
Web-based interventions for smoking cessation	4	6.2
Social media interventions for smoking cessation	3	4.6
Tailored/personalized interventions for smoking cessation	3	4.6
Virtual reality interventions for smoking cessation	2	3.1

1. Employing virtual reality interventions for smoking cessation [62, 63].
2. Implementing telemedicine/telehealth interventions for smoking cessation [64, 65].
3. Developing personalized/tailored interventions for smoking cessation [66, 67].
4. Addressing smoking cessation interventions for specific populations such as socioeconomically disadvantaged young adults, people living with HIV, cancer survivors, and veterans [68, 69].
5. Use of technology-based interventions in countries that are not in North America or Western Europe, such as Thailand, South Africa, Brazil, and Japan [70, 71].

## Discussion

The use of digital technology in the prevention and treatment of tobacco smoking has gained significant attention in recent years as indicated by the increase in the number of publications after 2011. The period after 2011 has witnessed a widespread adoption of smartphones, mobile applications, and wearable devices [72]. In the past two decades, there have been an increasing recognition of the importance of smoking as a risk factor for several fatal NCDs [73]. This have prompted researchers and funding bodies to search for an acceptable, effective, and relatively inexpensive method to curb smoking prevalence by promoting smoking cessation [74]. The fact that scholars from the US contributed to more than half of the retrieved articles, does not necessarily mean that the US had the highest prevalence of tobacco smoking. The research activity of any particular country depends on several factors that include governmental initiatives, research infrastructure, research environment, funding availability, and the prevalence or importance of the topic to national and global health [75, 76]. It is possible that the research output and country rank are linked to the extent of advancement and use of technology in that particular country. Countries with higher rates of technology adoption among their populations may be more open and willing to use technology-based intervention to manage tobacco smoking. The observation that China ranked seventh and India ranked eleventh holds profound significance. These rankings underscore the global impact of tobacco smoking, particularly in two of the most populous countries worldwide [77]. It implies the need for tailored and culturally sensitive technology-based strategies to address the unique challenges in these countries. Additionally, understanding these rankings provides valuable insights into the cultural and regional factors influencing smoking habits, enabling the development of contextually relevant interventions. These rankings allows for comparative analyses of China and India’s approaches to tobacco smoking prevention and treatment, offering lessons and best practices for global sharing. It also emphasizes the significance of research and investments in technology-based solutions to combat tobacco smoking challenges in these countries. Furthermore, policymakers can use this information to inform public health policies, taxation, and regulatory measures related to tobacco control. Lastly, tracking these rankings over time can reveal trends in tobacco use and the effectiveness of prevention and treatment strategies, facilitating ongoing assessments of various interventions’ impact. This explanation remains speculative and further investigations are needed to determine the factors affecting the research output in technology-based interventions in the general discipline of health.

The findings in the current study indicated that the vast majority of research activity was directed toward tobacco smoking treatment, specifically smoking cessation, while relatively limited research was published on technology-based prevention. The emphasis of technology-based research on smoking cessation rather than preventive strategies could be attributed to several factors. Quitting smoking is challenging to smokers and the use of technology offers a new avenue to support smoking cessation [78–81]. Research funding agencies prioritize research on smoking cessation interventions that produce immediate effects. Preventive strategies including awareness and educational campaigns could be achieved by non-traditional methods and their effects are seen on the long term rather than on the short term. A second reason for the greater emphasis on smoking cessation research is related to the relatively easier method to measure the outcome of smoking cessation using technology tools, which is not the case in measuring the outcome of prevention through policies and awareness campaigns. Tobacco smoking policies such as smoking-free places needs long times to show effects on smoking prevalence. The same applies to awareness campaigns that require years to show positive effects in contrast to smoking cessation.

Mapping of frequent terms showed the presence of a research theme related to smoking cessation among students, adolescents, and young adults. Adolescents are considered vulnerable when it comes to smoking initiation and addiction. Research has shown that the majority of adult smokers start smoking during their teenage years or yearly adulthood [82, 83]. Secondly, the familiarity of technology among young adults makes it easier to carry out research utilizing modern technology [84]. Finally, technology-based interventions have demonstrated promising results regarding effectiveness and reach among young populations. Technology-based interventions can offer personalized support, deliver tailored messages, provide interactive tools, and leverage social support networks, which are all factors that may be appealing and effective in engaging young individuals in quitting smoking [85–87].

Another important feature revealed by mapping frequent author keywords was the limited used of social media in the context of tobacco smoking and prevention. Several factors could explain this. Despite the very wide spread use of social media, there is limited evidence-based about the effectiveness of social media in smoking cessation. This makes research on social media-based tobacco smoking prevention and treatment limited compared to smartphone or internet-based methodology. A second possible reason for the limited research activity using social media is the regulations imposed by social media that restrict tobacco– related content material. This might limit the utilization of social media in tobacco-related research.

Analysis showed that the use of text messaging to help in smoking cessation constituted a research theme and articles on text messaging received relatively high number of citations. The use of text messaging as a tool for smoking cessation represents a relatively new and innovative approach in the field. The integration of text messaging as an intervention strategy offered a fresh perspective and attracted significant attention from both researchers and policy makers. Text messaging is currently a widely used means of communication among different groups of populations including smokers. The use of text misaligning tool capitalize on the prevalence and convenience of mobile phones, making it easier to reach and engage with smokers. The text messaging has the advantage of scalability, which makes it appealing for researchers and public health practitioners who aim to implement and measure the efficacy of cessation program on a large scale. The research findings consistently demonstrated positive results and effectiveness as measured by increasing smoking abstinence rates, acceptability, and feedback from users. These advantages of text messaging tools increased the interest of researchers in this technology-based tool. Finally, the majority of RCTs on smoking cessation showed promising and successful results of the text messaging tool, which adds to the credibility, scientific rigor, and reliability of the tool within the scientific committee [88–91]. Based on all the above, health policy makers should promote the use and monitoring of text messaging method in smoking cessation tool in primary healthcare services. It is expected that future research will witness greater research activity on the use of virtual reality and telemedicine in smoking cessation programs [62, 64, 65, 92]. Furthermore, personalized and tailored interventions in special population groups, such as cancer survivors, will witness greater research activity [66, 67].Future research activity using technology for tobacco smoking will not be limited to high-income countries. It is expected that research activity using technology-based tools in smoking cessation will be worldwide [37, 70, 93].

### Research gaps and future research directions

The research landscape analysis of technology-based tobacco smoking prevention and control gave an overall insight of has been published in this field. However, the research landscape also shows certain gaps and limitations in scientific research in this field. For example, most of the highly cited articles that had a positive impact on the field were limited by short follow up. Most of the articles had a follow up for several weeks to several months and there is a noticeable lack of long-term follow up investigations. Longer follow up times are needed to confirm sustainable effectiveness of the methods. Future research should focus on extended follow up assessments to confirm efficacy and relapse rates. Secondly, most studies, especially those with the high citations, included participants from high-income countries. This limits the generalizability of the conclusions of the studies. Future studies should have a diverse group of participants with different socioeconomic background, ethnic groups, gender, religious groups, and geographical distribution. The majority of studies testing the efficacy of the RCTs using technology-based tools have variable components, which makes comparison difficult. Therefore, it is recommended that future standardized method should be used to make comparison between various articles credible and reliable. Articles that reported benefit of technology-based tool in smoking cessation relied on self-reported abstinence, which is subject to recall bias and error [94, 95]. Therefore, future research should focus on biochemical measures to confirm abstinence. Future research should also focus on two major areas related to future implementation of technology in smoking cessation activities. Potential adverse health effects of technology methods and cost-effectiveness should be investigated and reported.

### Limitations

The current research landscape study was limited by the use of single database and by the possible presence of false-positive and false-negative results. In research landscape analysis, such limitations are common but does not affect the overall analysis and identification of research hotspots, research themes, research gaps, and potential future research focus. In our study, we primarily focused on the bibliometric analysis of research articles related to technology-based interventions for smoking cessation. While our analysis provides a comprehensive overview of the research landscape, it is essential to acknowledge that assessing real-world technology adoption and utilization falls outside the scope of a bibliometric analysis. However, we recognize the significance of this aspect and its potential relevance to the field. In practice, the diffusion of technology-based interventions for tobacco smoking prevention and treatment is influenced by various factors, including accessibility, acceptance, and adoption rates among users. We did not directly collect data on real-world utilization in this study. Nevertheless, the widespread availability of such interventions and their increasing integration into healthcare systems and public health campaigns suggest a growing interest in their real-world use. Future research endeavors may explore the adoption rates, user experiences, and effectiveness of these interventions, providing insights into their diffusion and practical impact on smoking cessation efforts.

## Conclusions

In this extensive bibliometric analysis spanning two decades (2003–2022), our study provides a comprehensive overview of the research landscape in the domain of technology-based interventions for tobacco smoking prevention and treatment. The findings and insights obtained from this analysis offer a wealth of information that holds significant implications for the advancement of smoking cessation strategies and the future of public health. Several key takeaways emerge from this research landscape analysis. The exponential growth in research publications following 2011 corresponds with the widespread adoption of smartphones, mobile applications, and wearable devices. The emergence of these technologies has created unprecedented opportunities for innovative interventions in the field of tobacco smoking prevention and treatment. The leading role of the United States in contributing to more than half of the retrieved articles emphasizes the significance of technological advancement and its impact on research activity. It is essential to note that high research output does not necessarily reflect high smoking prevalence but rather indicates the extent of technological integration and investment in the research domain. The remarkable rankings of China and India as the seventh and eleventh countries, respectively, highlight the global reach and consequences of tobacco smoking, particularly in two of the most populous nations worldwide. This underscores the need for culturally tailored, context-specific technology-based solutions to address the distinct challenges these countries face. The overwhelming emphasis on smoking cessation research rather than prevention could be attributed to the immediate and measurable outcomes of cessation interventions. Moreover, the application of technology in smoking cessation provides a novel and effective avenue for supporting smokers in their quit attempts. The research landscape further reveals the critical role of text messaging as a promising tool for smoking cessation. The scalability, reach, and effectiveness of text messaging make it a compelling choice for researchers and policymakers alike. The presence of limited research on social media in the context of tobacco smoking prevention may be attributed to the limited evidence-based knowledge regarding the effectiveness of social media in this area, as well as the regulations imposed by social media platforms on tobacco-related content. To bridge existing research gaps, future studies should include long-term follow-up assessments to confirm the sustainability of intervention efficacy and expand participant diversity to improve the generalizability of findings. Standardized methods and biochemical measures to confirm abstinence should be incorporated in future research. Additionally, the investigation of potential adverse health effects and cost-effectiveness of technology-based methods in smoking cessation activities will be essential for comprehensive insights. This research landscape analysis serves as a valuable resource for researchers, healthcare professionals, policymakers, and public health advocates. The identified research hotspots, research themes, and emerging topics can guide the development of evidence-based interventions in technology-based tobacco smoking prevention and treatment. By continually monitoring the research landscape, stakeholders can track the evolving trends, assess the effectiveness of various interventions, and adapt strategies to combat the global tobacco smoking epidemic more effectively. In conclusion, technology-based interventions have shown great promise in supporting smoking cessation and reducing relapse rates. As technology continues to advance and integrate further into our lives, it is evident that these interventions will play a pivotal role in tobacco control efforts. This comprehensive analysis underscores the critical importance of technology in public health strategies and highlights the vast potential for innovative, evidence-based approaches to mitigate the adverse health effects of tobacco smoking worldwide. Future research, innovation, and collaboration will drive the continued evolution of technology-based interventions and pave the way for a smoke-free future.

### Electronic supplementary material

Below is the link to the electronic supplementary material.

Supplement 1: Keywords used to retrieve articles on technology-based interventions for tobacco smoking prevention and treatment.

## Data Availability

The datasets generated and/or analyzed during the current study are available in the Scopus repository, (www.scopus.com).

## References

[1] World Health Organization (WHO). WHO Framework Convention on Tobacco Control (WHO FCTC): WHO; Available from: WHO Framework Convention on Tobacco Control (WHO FCTC).

[2] Picciotto MR, Kenny PJ. Mechanisms of Nicotine Addiction. Cold Spring Harb Perspect Med. 2021;11(5). 10.1101/cshperspect.a039610. 10.1101/cshperspect.a039610 PMC8091956 32341069

[3] Prochaska JJ, Benowitz NL (2019). Current advances in research in treatment and recovery: nicotine addiction. Sci Adv.

[4] Kashiwabara M, Armada F (2013). Mind your smoking manners: the tobacco industry tactics to normalize smoking in Japan. Kobe J Med Sci.

[5] Robertson L, McGee R, Marsh L, Hoek J (2015). A systematic review on the impact of point-of-sale tobacco promotion on smoking. Nicotine Tob Res.

[6] Yong HH, Chow R, East K, Thrasher JF, Hitchman SC, Borland R (2023). Do social norms for cigarette smoking and nicotine Vaping Product Use Predict trying Nicotine Vaping products and attempts to quit cigarette smoking amongst adult smokers? Findings from the 2016–2020 International Tobacco Control Four Country smoking and vaping surveys. Nicotine Tob Res.

[7] Sansone N, Yong HH, Li L, Jiang Y, Fong GT (2015). Perceived acceptability of female smoking in China. Tob Control.

[8] Rocha SAV, Hoepers ATC, Fröde TS, Steidle LJM, Pizzichini E, Pizzichini MMM (2019). Prevalence of smoking and reasons for continuing to smoke: a population-based study. J Bras Pneumol.

[9] Fiatal S, Tóth R, Moravcsik-Kornyicki Á, Kósa Z, Sándor J, McKee M (2016). High prevalence of smoking in the Roma Population seems to have no genetic background. Nicotine Tob Res.

[10] Wills L, Kenny PJ (2021). Addiction-related neuroadaptations following chronic nicotine exposure. J Neurochem.

[11] Levy DT, Tam J, Kuo C, Fong GT, Chaloupka F (2018). The impact of implementing Tobacco Control policies: the 2017 Tobacco Control Policy Scorecard. J Public Health Manag Pract.

[12] Litvin EB, Abrantes AM, Brown RA (2013). Computer and mobile technology-based interventions for substance use disorders: an organizing framework. Addict Behav.

[13] Sugarman DE, Campbell ANC, Iles BR, Greenfield SF (2017). Technology-based interventions for Substance Use and Comorbid disorders: an examination of the emerging literature. Harv Rev Psychiatry.

[14] Fadus MC, Squeglia LM, Valadez EA, Tomko RL, Bryant BE, Gray KM (2019). Adolescent substance Use Disorder Treatment: an update on evidence-based strategies. Curr Psychiatry Rep.

[15] Soyka M, Mutschler J (2016). Treatment-refractory substance use disorder: focus on alcohol, opioids, and cocaine. Prog Neuropsychopharmacol Biol Psychiatry.

[16] Curtis BL, Ashford RD, Magnuson KI, Ryan-Pettes SR (2019). Comparison of Smartphone Ownership, Social Media Use, and willingness to Use Digital interventions between Generation Z and millennials in the treatment of Substance Use: cross-sectional questionnaire study. J Med Internet Res.

[17] Hsu M, Martin B, Ahmed S, Torous J, Suzuki J (2022). Smartphone ownership, Smartphone Utilization, and Interest in Using Mental Health Apps to address Substance Use disorders: Literature Review and cross-sectional Survey Study Across two sites. JMIR Form Res.

[18] Prutzman YM, Wiseman KP, Grady MA, Budenz A, Grenen EG, Vercammen LK et al. (2021): Using Digital Technologies to Reach Tobacco Users Who Want to Quit: Evidence From the National Cancer Institute’s Smokefree.gov Initiative. American Journal of Preventive Medicine 60(3, Supplement 2):S172-S84. 10.1016/j.amepre.2020.08.008. 10.1016/j.amepre.2020.08.008 33663705

[19] Liu L, Zhao Y, Li J, Zhang N, Lan Z, Liu X (2023). Efficacy of digital therapeutics in smoking cessation: a systematic review and meta-analysis. Med Novel Technol Devices.

[20] Micalizzi L, Mattingly DT, Hart JL, Jensen JK, Mahabee-Gittens EM, Garrison KA (2023). Smartphone Apps Targeting Youth Tobacco Use Prevention and Cessation: an Assessment of credibility and quality. Curr Addict Rep.

[21] Cobos-Campos R, Mar J, Apiñaniz A, de Lafuente AS, Parraza N, Aizpuru F (2021). Cost-effectiveness analysis of text messaging to support health advice for smoking cessation. Cost Eff Resource Allocation.

[22] Allan TD, Ashish AD, Damon JV, Alexander VP, Summer GF, Patricia DT (2019). Cost-effectiveness analysis of smoking cessation interventions using cell phones in a low-income population. Tob Control.

[23] Chu K-H, Matheny SJ, Escobar-Viera CG, Wessel C, Notier AE, Davis EM (2021). Smartphone health apps for tobacco Cessation: a systematic review. Addict Behav.

[24] World Health Organization (WHO). Wacfh, safer lives. Who we are: Available from: https://www.who.int/about/who-we-are.

[25] Centers for Disease Prevention and Control (CDC). Saving Lives, Protecting People ^TM^ Available from: https://www.cdc.gov/about/index.html.

[26] Arruda H, Silva ER, Lessa M, Proença D, Bartholo R (2022). VOSviewer and Bibliometrix. J Med Libr Assoc.

[27] van Eck NJ, Waltman L (2010). Software survey: VOSviewer, a computer program for bibliometric mapping. Scientometrics.

[28] Cheng F, Xu J, Su C, Fu X, Bricker J (2017). Content Analysis of Smartphone Apps for Smoking Cessation in China: empirical study. JMIR Mhealth Uhealth.

[29] Robinson CD, Seaman EL, Grenen E, Montgomery L, Yockey RA, Coa K (2020). A content analysis of smartphone apps for adolescent smoking cessation. Transl Behav Med.

[30] Graham AL, Papandonatos GD, Cha S, Amato MS, Jacobs MA, Cohn AM (2022). Effectiveness of an optimized text message and internet intervention for smoking cessation: a randomized controlled trial. Addiction.

[31] Lin H, Li X, Zhang Y, Wen Z, Guo Z, Yang Y (2023). A randomized controlled trial of personalized text messages for smoking cessation, China. Bull World Health Organ.

[32] Bricker JB, Watson NL, Heffner JL, Sullivan B, Mull K, Kwon D (2020). A smartphone app designed to Help Cancer patients Stop Smoking: results from a pilot randomized trial on feasibility, acceptability, and effectiveness. JMIR Form Res.

[33] Rostami M, Moheban F, Davoudi M, Heshmati K, Taheri AA (2022). Current Status and Future trends of Acceptance and Commitment Therapy (ACT) for Smoking Cessation: a narrative review with specific attention to technology-based interventions. Addict Health.

[34] Santiago-Torres M, Mull KE, Sullivan BM, Kwon D, Nollen NL, Zvolensky MJ (2022). Efficacy and utilization of an acceptance and commitment therapy-based smartphone application for smoking cessation among black adults: secondary analysis of the iCanQuit randomized trial. Addiction.

[35] Santiago-Torres M, Mull KE, Sullivan BM, Rigotti NA, Bricker JB (2023). Acceptance and Commitment Therapy-based Smartphone Applications for Cessation of Tobacco Use among adults with high nicotine dependence: results from the iCanQuit Randomized Trial. Subst Use Misuse.

[36] Haug S, Castro RP, Kwon M, Filler A, Kowatsch T, Schaub MP (2015). Smartphone use and smartphone addiction among young people in Switzerland. J Behav Addict.

[37] Chulasai P, Chinwong D, Vientong P, Lertsinudom S, Kanjanarat P, Hall JJ, et al. Smartphone application for Smoking Cessation (quit with US): a randomized controlled trial among young adult light smokers in Thailand. Int J Environ Res Public Health. 2022;19(14). 10.3390/ijerph19148265. 10.3390/ijerph19148265 PMC9321212 35886120

[38] Bendtsen M (2020). Heterogeneous treatment effects of a text messaging smoking cessation intervention among university students. PLoS ONE.

[39] Shuter J, Chander G, Graham AL, Kim RS, Stanton CA (2022). Randomized Trial of a web-based Tobacco Treatment and Online Community support for people with HIV attempting to quit smoking cigarettes. J Acquir Immune Defic Syndr.

[40] Schnall R, Liu J, Alvarez G, Porras T, Ganzhorn S, Boerner S, et al. A smoking Cessation Mobile app for persons living with HIV: preliminary efficacy and feasibility study. JMIR Formative Research. 2022;6(8). 10.2196/28626. 10.2196/28626 PMC9437787 35980739

[41] do Amaral LM, Ronzani TM, Cruvinel E, Richter K, Oliveira Andrade R, Lanzieri IO, et al. Text messaging interventions to support smoking cessation among hospitalized patients in Brazil: a randomized comparative effectiveness clinical trial. BMC Res Notes. 2022;15(1). 10.1186/s13104-022-06002-6. 10.1186/s13104-022-06002-6 PMC8962029 35346351

[42] McDuffie AC, Varughese SJ, Duffy AR, Faiella AS, Wegener LF, Singer KA (2022). Pharmacist-led telehealth tobacco cessation services compared with usual care in a community health center. J Am Pharmacists Association.

[43] Saul JE, Amato MS, Cha S, Graham AL (2016). Engagement and attrition in internet smoking cessation interventions: insights from a cross-sectional survey of one-hit-wonders. Internet Interventions.

[44] Strecher VJ, McClure J, Alexander G, Chakraborty B, Nair V, Konkel J, et al. The role of engagement in a tailored web-based smoking cessation program: Randomized controlled trial. J Med Internet Res. 2008;10(5). 10.2196/jmir.1002. 10.2196/jmir.1002 PMC2630833 18984557

[45] Thrul J, Klein AB, Ramo DE. Smoking cessation intervention on facebook: which content generates the best engagement? J Med Internet Res. 2015;17(11). 10.2196/jmir.4575. 10.2196/jmir.4575 PMC4704894 26561529

[46] Tanigawa T, Nomura A, Kuroda M, Muto T, Hida E, Satake K. Comparing telemedicine and face-to-face consultation based on the standard smoking cessation program for nicotine dependence: protocol for a randomized controlled trial. JMIR Res Protocols. 2019;8(7). 10.2196/12701. 10.2196/12701 PMC6647761 31290402

[47] Vidrine JI, Shih YCT, Businelle MS, Sutton SK, Hoover DS, Cottrell-Daniels C, et al. Comparison of an automated smartphone-based smoking cessation intervention versus standard quitline-delivered treatment among underserved smokers: protocol for a randomized controlled trial. BMC Public Health. 2022;22(1). 10.1186/s12889-022-12840-7. 10.1186/s12889-022-12840-7 PMC8939152 35317789

[48] Daly AT, Deshmukh AA, Vidrine DJ, Prokhorov AV, Frank SG, Tahay PD (2019). Cost-effectiveness analysis of smoking cessation interventions using cell phones in a low-income population. Tob Control.

[49] Mujcic A, Blankers M, Boon B, Verdonck-De Leeuw IM, Smit F, Van Laar M, et al. Effectiveness, Cost-effectiveness, and cost-utility of a Digital Smoking Cessation intervention for Cancer survivors: Health Economic evaluation and outcomes of a pragmatic randomized controlled trial. J Med Internet Res. 2022;24(3). 10.2196/27588. 10.2196/27588 PMC9491833 35297777

[50] Whitemore R, Leonardi-Bee J, Naughton F, Sutton S, Cooper S, Parrott S, et al. Effectiveness and cost-effectiveness of a tailored text-message programme (MiQuit) for smoking cessation in pregnancy: study protocol for a randomised controlled trial (RCT) and meta-analysis. Trials. 2019;20(1). 10.1186/s13063-019-3341-4. 10.1186/s13063-019-3341-4 PMC6530023 31118090

[51] Brendryen H, Kraft P (2008). Happy ending: a randomized controlled trial of a digital multi-media smoking cessation intervention. Addiction.

[52] Strecher VJ, Shiffman S, West R (2005). Randomized controlled trial of a web-based computer-tailored smoking cessation program as a supplement to nicotine patch therapy. Addiction.

[53] Abroms LC, Lee Westmaas J, Bontemps-Jones J, Ramani R, Mellerson J (2013). A content analysis of popular smartphone apps for smoking cessation. Am J Prev Med.

[54] Strecher VJ, McClure JB, Alexander GL, Chakraborty B, Nair VN, Konkel JM (2008). Web-based Smoking-Cessation Programs. Results of a Randomized Trial. Am J Prev Med.

[55] Bricker JB, Mull KE, Kientz JA, Vilardaga R, Mercer LD, Akioka KJ (2014). Randomized, controlled pilot trial of a smartphone app for smoking cessation using acceptance and commitment therapy. Drug Alcohol Depend.

[56] Obermayer JL, Riley WT, Asif O, Jean-Mary J (2004). College smoking-cessation using cell phone text messaging. J Am Coll Health.

[57] Free C, Knight R, Robertson S, Whittaker R, Edwards P, Zhou W (2011). Smoking cessation support delivered via mobile phone text messaging (txt2stop): a single-blind, randomised trial. The Lancet.

[58] Cobb NK, Graham AL, Bock BC, Papandonatos G, Abrams DB (2005). Initial evaluation of a real-world internet smoking cessation system. Nicotine and Tobacco Research.

[59] Rodgers A, Corbett T, Bramley D, Riddell T, Wills M, Lin RB (2005). Do u smoke after txt? Results of a randomised trial of smoking cessation using mobile phone text messaging. Tob Control.

[60] Strecher VJ, McClure J, Alexander G, Chakraborty B, Nair V, Konkel J (2008). The role of engagement in a tailored web-based smoking cessation program: randomized controlled trial. J Med Internet Res.

[61] Cobb NK, Graham AL, Bock BC, Papandonatos G, Abrams DB (2005). Initial evaluation of a real-world internet smoking cessation system. Nicotine Tob Res.

[62] Borrelli B, Endrighi R, Jurasic MM, Hernandez H, Jones E, Ospina J (2022). A smoking cessation induction intervention via virtual reality headset during a dental cleaning: protocol for a randomized controlled trial. BMC Public Health.

[63] Borrelli B, Rueras N, Jurasic M (2021). Delivery of a smoking cessation induction intervention via virtual reality headset during a dental cleaning. Transl Behav Med.

[64] Nomura A, Tanigawa T, Muto T, Oga T, Fukushima Y, Kiyosue A (2019). Clinical efficacy of Telemedicine compared to face-to-face Clinic visits for Smoking Cessation: Multicenter open-label Randomized Controlled Noninferiority Trial. J Med Internet Res.

[65] Spears CA, Mhende J, Hawkins C, Do VV, Hayat MJ, Eriksen MP (2022). Mindfulness-based Smoking Cessation Delivered through Telehealth and text messaging for low-income smokers: protocol for a Randomized Controlled Trial. JMIR Res Protoc.

[66] Pratt R, Ojo-Fati O, Adam A, Sharif H, Kahin A, Mahamud A (2020). Text message support for Smoking Cessation during Ramadan: a Focus Group Study with Somali immigrant Muslim men. Nicotine Tob Res.

[67] Villanti AC, Peasley-Miklus C, Cha S, Schulz J, Klemperer EM, LePine SE (2022). Tailored text message and web intervention for smoking cessation in U.S. socioeconomically-disadvantaged young adults: a randomized controlled trial. Prev Med.

[68] Asfar T, Alcaide ML, Jones DL, McClure LA, Brewer J, Lee DJ (2022). HIV patients’ perceptions of a potential multi-component mindfulness-based smoking cessation smartphone application intervention. PLoS ONE.

[69] Bui TC, Sopheab H, Businelle MS, Chhea C, Ly SP, Vidrine JI (2022). Mobile-health intervention for smoking cessation among Cambodian people living with HIV: a mixed-methods pilot study. AIDS Care.

[70] Alqahtani AS (2022). Awareness of current mobile apps for smoking cessation among the dental and medical practitioners in Saudi Arabia. Eur Rev Med Pharmacol Sci.

[71] BinDhim NF, McGeechan K, Trevena L (2018). Smartphone Smoking Cessation application (SSC app) trial: a multicountry double-blind automated randomised controlled trial of a smoking cessation decision-aid ‘app’. BMJ Open.

[72] Statista. Number of smartphone mobile network subscriptions worldwide from 2016 to 2022, with forecasts from 2023 to 2028 2023 Available from: https://www.statista.com/statistics/330695/number-of-smartphone-users-worldwide/.

[73] Centers for Disease Control and Prevention (CDC). Smoking and Tobacco use (Health effects) Available from: https://www.cdc.gov/tobacco/basic_information/health_effects/index.htm#:~:text=Smoking%20causes%20cancer%2C%20heart%20disease,immune%20system%2C%20including%20rheumatoid%20arthritis.

[74] World Health Organization (WHO). WHO global report on trends in prevalence of tobacco use 2000–2025 2019 [Third edition:[Available from: https://www.who.int/publications/i/item/who-global-report-on-trends-in-prevalence-of-tobacco-use-2000-2025-third-edition.

[75] Lavis JN, Lomas J, Hamid M, Sewankambo NK (2006). Assessing country-level efforts to link research to action. Bull World Health Organ.

[76] Schmutz A, Salignat C, Plotkina D, Devouassoux A, Lee T, Arnold M (2019). Mapping the Global Cancer Research Funding Landscape. JNCI Cancer Spectr.

[77] Worldometer. Countries in the world by population 2023 Available from: https://www.worldometers.info/world-population/population-by-country/.

[78] McKelvey K, Thrul J, Ramo D (2017). Impact of quitting smoking and smoking cessation treatment on substance use outcomes: an updated and narrative review. Addict Behav.

[79] Collins GB, Jerry JM, Bales R (2015). Quitting smoking: still a challenge, but newer tools show promise. Cleve Clin J Med.

[80] Komiyama M, Takahashi Y, Tateno H, Mori M, Nagayoshi N, Yonehara H (2019). Support for patients who have difficulty quitting smoking: a review. Intern Med.

[81] Babb S, Malarcher A, Schauer G, Asman K, Jamal A (2017). Quitting smoking among adults - United States, 2000–2015. MMWR Morb Mortal Wkly Rep.

[82] Chassin L, Presson CC, Rose JS, Sherman SJ (1996). The natural history of cigarette smoking from adolescence to adulthood: demographic predictors of continuity and change. Health Psychol.

[83] Gilman SE, Rende R, Boergers J, Abrams DB, Buka SL, Clark MA (2009). Parental smoking and adolescent smoking initiation: an intergenerational perspective on tobacco control. Pediatrics.

[84] Pew Research Center. Teens and Technology. 2013 Available from: https://www.pewresearch.org/internet/2013/03/13/teens-and-technology-2013/.

[85] Free C, Knight R, Robertson S, Whittaker R, Edwards P, Zhou W (2011). Smoking cessation support delivered via mobile phone text messaging (txt2stop): a single-blind, randomised trial. Lancet.

[86] Abroms LC, Boal AL, Simmens SJ, Mendel JA, Windsor RA (2014). A randomized trial of Text2Quit: a text messaging program for smoking cessation. Am J Prev Med.

[87] Ubhi HK, Michie S, Kotz D, Wong WC, West R (2015). A mobile app to aid smoking cessation: preliminary evaluation of SmokeFree28. J Med Internet Res.

[88] Kong G, Ells DM, Camenga DR, Krishnan-Sarin S (2014). Text messaging-based smoking cessation intervention: a narrative review. Addict Behav.

[89] Whittaker R, McRobbie H, Bullen C, Rodgers A, Gu Y, Dobson R (2019). Mobile phone text messaging and app-based interventions for smoking cessation. Cochrane Database Syst Rev.

[90] Balmumcu A, Ünsal Atan Ş (2021). Smoking Cessation Programs for pregnant women: utilizing WhatsApp text messaging. J Addict Nurs.

[91] Budenz A, Coa K, Grenen E, Keefe B, Sanders A, Wiseman KP (2022). User experiences with an SMS text Messaging Program for Smoking Cessation: qualitative study. JMIR Form Res.

[92] Keijsers M, Vega-Corredor MC, Tomintz M, Hoermann S (2021). Virtual reality technology use in cigarette craving and smoking interventions (I virtually quit): systematic review. J Med Internet Res.

[93] Formagini TD, Ervilha RR, Machado NM, Andrade BA, Gomide HP, Ronzani TM (2017). A review of smartphone apps for smoking cessation available in Portuguese. Cad Saude Publica.

[94] Randomized Trial of a Smartphone Mobile Application compared to text messaging to Support Smoking Cessation. Telemedicine and e-Health (2014);20(3):206–14. 10.1089/tmj.2013.0169. 10.1089/tmj.2013.0169 PMC3934597 24350804

[95] Liao Y, Wu Q, Tang J, Zhang F, Wang X, Qi C (2016). The efficacy of mobile phone-based text message interventions (‘Happy quit’) for smoking cessation in China. BMC Public Health.

