# Supplementary material for: Technology-based interventions for tobacco smoking prevention and treatment: a 20-year bibliometric analysis (2003–2022)
Source: Subst Abuse Treat Prev Policy. 2024 Feb 6;19:13. doi: 10.1186/s13011-024-00595-w (PMC10848402; doi:10.1186/s13011-024-00595-w)
Supplement: Supplementary file 1 — Supplement 1: Keywords used to retrieve articles on technology-based interventions for tobacco smoking prevention and treatment. [file 13011_2024_595_MOESM1_ESM.doc]

**Technology-based interventions for tobacco smoking prevention and treatment: A 20-year bibliometric analysis (2003 – 2022)**

**Supplement 1**

**Keywords** used to retrieve relevant articles on technology-based interventions for tobacco smoking prevention and treatment

1. **Keywords related to tobacco smoking**

smoking or tobacco or "tobacco use" or "tobacco consumption" or "anti-smoking"

1. **Keywords related to technology and social media**

twitter or facebook or whatsapp or "social media" or "e-mail" or telehealth or "mobile app*" or "cell phone" or "online program" or "computer-assisted" or "computer tailored" or "mobile phone" or "technology-based" or "digital" or "e-health" or "mobile health" or "mhealth" or "web-based" or ( internet and intervention and cessation ) or ( internet and program and cessation ) or ( internet and trial and cessation ) or ( internet and system and cessation ) or "computer-based" or "telehealth" or telemedicine or "virtual reality" or "electronic interventions" or "smartphone" or "online smoking cessation" or "text messag*" or "wearable device" or "social media" or "gamification" or "internet-based" or ( digital and media ) or ( digital and program ) or "on line system".

1. **Keywords related to prevention and treatment in the context of tobacco smoking**

stop or restriction or prevent* or treatment or cessation or quit or intervention or therapy or "health promotion" or awareness or education or campaign or advertising or help or detox*

1. **Filters**

1, Only research articles published in peer-reviewed journals were included. Therefore, all types of reviews, letters, editorials, notes, errata, books, book chapters, and conferences were excluded

2. Time limitation: 2003 – 2022. Any article published before 2003 or after 2022 were excluded.

3. Language: articles with non-English abstracts were excluded

4. Articles on e-cigarettes/electronic cigarettes were excluded because the focus was on traditional tobacco smoking.

Articles with the following terms in the title were excluded: training, advice, opioids

1. **Search strategy: All terms employed in the search strategy were used in the “TITLE” search to decrease the number of false-positive and sharpen the results.**
